# A spline-based regression parameter set for creating customized DARTEL MRI brain templates from infancy to old age

**DOI:** 10.1016/j.dib.2017.12.001

**Published:** 2017-12-12

**Authors:** Marko Wilke

**Affiliations:** Department of Pediatric Neurology and Developmental Medicine, Children's Hospital and Experimental Pediatric Neuroimaging group, Children's Hospital & Dept. of Neuroradiology, University of Tübingen, Germany

**Keywords:** MRI template creation, Multivariate adaptive regression splines, DARTEL, Structural MRI

## Abstract

This dataset contains the regression parameters derived by analyzing segmented brain MRI images (gray matter and white matter) from a large population of healthy subjects, using a multivariate adaptive regression splines approach. A total of 1919 MRI datasets ranging in age from 1–75 years from four publicly available datasets (NIH, C-MIND, fCONN, and IXI) were segmented using the CAT12 segmentation framework, writing out gray matter and white matter images normalized using an affine-only spatial normalization approach. These images were then subjected to a six-step DARTEL procedure, employing an iterative non-linear registration approach and yielding increasingly crisp intermediate images. The resulting six datasets per tissue class were then analyzed using multivariate adaptive regression splines, using the CerebroMatic toolbox. This approach allows for flexibly modelling smoothly varying trajectories while taking into account demographic (age, gender) as well as technical (field strength, data quality) predictors. The resulting regression parameters described here can be used to generate matched DARTEL or SHOOT templates for a given population under study, from infancy to old age. The dataset and the algorithm used to generate it are publicly available at https://irc.cchmc.org/software/cerebromatic.php.

**Specifications Table**

**Table:** 

Subject area	Neuroscience
More specific subject area	Computational Neuroscience
Type of data	Statistical regression parameter set
How data was acquired	This dataset is based on high-resolution T1 3D structural MR imaging data of 1919 subjects acquired at 1.5 and 3 T from four publicly available datasets (NIH, C-MIND, fCONN, and IXI)
Data format	Analyzed dataset in Matlab ® datafile format (*.mat, v7.3)
Experimental factors	While employing a multivariate adaptive regression splines, demographic (age and gender) as well as technical (field strength and data quality) factors were taken into account
Experimental features	Images were segmented using the CAT12 toolbox and spatially normalized therein using an affine-only spatial normalization approach
Data source location	All source data is available from the contributing studies (NIH, C-MIND, fCONN, and IXI) at their respective websites
Data accessibility	The dataset as well as the algorithms used are freely available at https://irc.cchmc.org/software/cerebromatic.php

**Value of the data**

• Segmentation and spatial normalization of brain MR imaging data routinely makes use of reference, or template brains, which have to be appropriate for the dataset under study
• Instead of simply averaging participant's data, template creation can also be achieved using statistical regression approaches, which allow for taking into account key demographic and technical predictors of the dataset
• For high-dimensional warping approaches such as the popular DARTEL or SHOOT algorithm, a large population is needed to create high-quality templates, which is not always available especially for “unusual” populations such as infants and older participants
• This dataset is the result of analyzing a large population of healthy subjects using a multivariate adaptive regression splines approach, allowing for the customized creation of high-quality sets of brain templates to be used within the DARTEL/SHOOT framework
• Such externally-generated but matched templates are particularly useful when only a small and/or “unusual” dataset is available for study

## 1 Data

This regression parameter dataset is based on high-resolution T1 3D structural brain MR imaging data of 1919 healthy subjects aged 13–900 months [1–75 years]. Images were acquired at 1.5 and 3 T and were selected from four publicly available datasets (NIH, C-MIND, fCONN, and IXI). The dataset contains regression parameters from 6 DARTEL iterations for GM and WM each and can be used within the CerebroMatic toolbox to generate matched DARTEL/SHOOT templates for a researcher's own population.

## 2 Experimental design

**Fig. 1:**
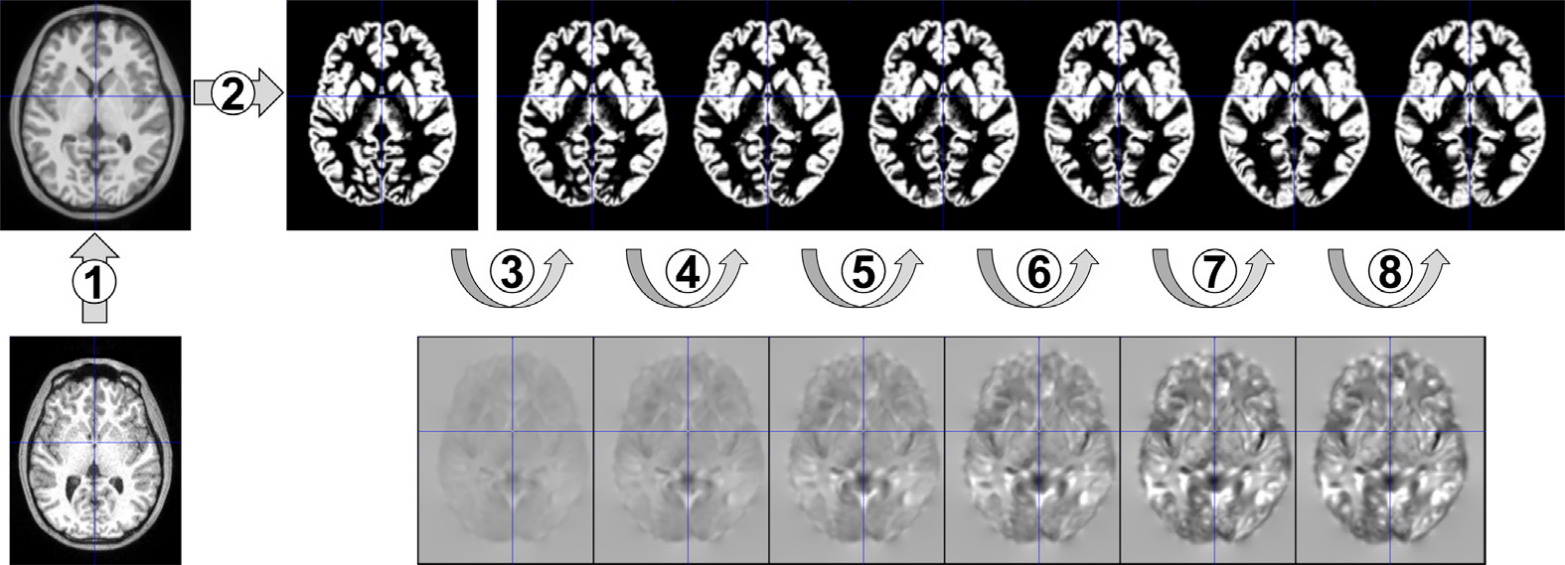
Overview of the intermediate steps of the image data processing pipeline: each whole brain T1 3D datasets was first bias-corrected (1) and, using CAT12, segmented into GM (2) and WM (not shown). Then, an iterative non-linear registration (3–8) to the respective group mean tissue map was applied, resulting in ever crisper tissue maps (upper row) and corresponding deformation fields (lower row, illustrated here by their Jacobian determinants). This results in conventional DARTEL templates (see Fig. 2, Fig. 4, top rows). The tissue maps were also submitted to the CerebroMatic toolbox, resulting in synthetic DARTEL templates (see Fig. 2, Fig. 4, bottom rows).

The aim of this article is to describe a set of regression parameters which can be used within the CerebroMatic toolbox [1]. The general approach of this toolbox is as follows: instead of simple averaging a large number of subject's brain MRI data (usually following tissue segmentation) to generate a reference brain/template, the data is instead analyzed statistically. The main advantage is that this approach is able to take into account the dominating demographic (such as age and gender [2]) and technical factors (such as field strength and data quality [3], [4]). As opposed to the previous application of this idea (and its implementation within the Template-O-Matic toolbox [2]), the CerebroMatic now uses a much more flexible statistical approach, namely multivariate adaptive regression splines [5]. This allows modeling smooth trajectories of change with much higher flexibility and accuracy, especially in the context of an inhomogeneous group (see [1], Fig. 1, for an illustration). The result of this modeling is a regression parameter set for each voxel, and each tissue class. From these parameters, a synthetic tissue class can then be generated as the predicted values are linear combinations of the original response values. Hence, the resulting tissue class can be described based on (and thus, matched to) the demographics of a new and independent input population.

A shortcoming of this approach, however, was that more current (and computationally intense) high-dimensional warping approaches such as the commonly-employed DARTEL- [6] or SHOOT-approach [7] use an iterative self-registration scheme. Hence, a single tissue prior is not appropriate anymore as these approaches require an increasingly crisp set of tissue priors to register to. The DARTEL-approach has shown great accuracy when compared with other non-linear spatial deformation approaches [8] and was later refined [7].^1^ To generate such high-quality tissue maps, however, large populations are required [1] which may not always be available, especially in the case of an “unusual” population such as children or elderly subjects. The here-described parameter set is the result of using the CerebroMatic toolbox to statistically generate such tissue prior sets for ultimate use within the DARTEL/SHOOT framework, based on a large population of healthy infants, children, and young as well as older adults.

## 3 Subjects and methods

For this data in brief article, the same initial datasets as already described in [1] were used, all of which are available from public repositories. Four large datasets with rigorous quality control mechanisms were selected, two for imaging data from children (the National Institute of Health's Study of Normal Brain Development [10] and the Cincinnati MR Imaging of Neurodevelopment study [11]) and two for imaging data from adults (The 1000 functional connectome study [12] and The Information eXtraction from Images study [13]). Following additional local quality control, a total of 1919 high-resolution 3D T1 images could be included. See Table 1 for demographic and imaging details of all included subjects. Further details on all subjects as well as respective credits, sponsors, and disclaimers can be found in the Supplementary material S1.

**Table 1:** Demographic information about all 4 contributing and the full dataset; *n*=number; T=Tesla. Values are described as sums or mean±SD. See text for details.

	***n***	**Age [months]**	**Voxel volume [µl]**	**Image quality [%]**	**1.5** **T [n]**	**3** **T [n]**
**NIH**	414	122.41±52.15	0.99±.08	76.47±10.39	414	0
**C-MIND**	206	99.87±55.8	0.99±.06	79.23±9.27	0	206
**fCONN**	757	331.76±156.35	1.18±.32	82.72±2.87	15	742
**IXI**	542	571.84±187.54	1.05±.03	83.81±1.98	178	364
**Full Dataset**	**1919**	**329.51**±**228.03**	**1.08±.22**	**81.31±6.7**	**607**	**1312**

Image data preprocessing was described in detail in [1] and is therefore only briefly summarized here. All data processing and analysis steps were performed in Matlab (Mathworks, Natick, MA), in part using functionality available within the spm12 software package (rev. 6906; University College London, UK). A 7th degree B-spline interpolation algorithm was used when writing images [14], but all other parameters were left at their default values unless specified otherwise. Initially, all images were reoriented and bias-corrected, using functionality provided within the unified segmentation framework [15]. Tissue segmentation was then achieved using the cat12 toolbox (r1092 [16]) which is a priorless modification and extension of the SPM12 “new segment” approach [17]. Tissue probability maps (for gray matter [GM] and white matter [WM] only) were spatially normalized using an affine registration scheme [18] to allow for an initial overlap of large structures. We opted for an affine approach here (instead of the usually recommended rigid-body procedure [6]) as the overall size difference between the subjects included here (between infancy and old age [1], [19]) must be expected to otherwise pose insurmountable challenges for the ensuing non-linear deformation steps (see below). Visual quality control was also performed as previously described [1], using individual inspection of each map at the level of the basal ganglia and the cerebellum to identify overt failure of spatial normalization or tissue segmentation.

The DARTEL approach performs an iterative but highly integrated spatial normalization scheme, in that all images in a population initially contribute to a straight mean to which then again all images are iteratively registered to. The images resulting from this first round are then again used to create a second average image, to which the images are again registered, and so on. Hence, in a first step, the standard DARTEL procedure (SPM12 batch module “DARTEL, create template”) was applied to the full dataset, yielding an initial set of six conventional templates for GM and WM each. In a second step (SPM12 batch module “Run DARTEL with existing template”), all images were then iteratively registered to these initial templates. However, the intermediate steps (reflecting the registration of each individual image to the first, second, third… template from the first step) are only computed internally, iteratively building on the results from the previous step. In order to obtain these intermediate images, the second processing job was therefore split into six successive jobs. The settings used correspond to the defaults and are listed in Table 2. After completing each iteration, the resulting intermediate deformation fields were copied before they were updated in the next iteration. See Fig. 1 for an overview. This ensures that each iteration builds upon the results from the previous step, in line with the original DARTEL approach. These twelve sets of deformation fields (two tissue classes per subject, times six iterations) were then used to write out corresponding sets of increasingly crisp tissue probability maps, six sets for GM and six sets for WM. These twelve sets of 1919 images each were then submitted for data analysis.

**Table 2:** DARTEL processing options used for each iteration. The penalizing energy term (linear elastic energy), the number of inner iterations (3), the Levenberg-Marquardt regularization (0.01), the number of cycles for the full multi-grid matrix solver (3) and the number of relaxations in each multi-grid cycle (3) as well as the third regularization parameter id (0.000001) were kept constant over iterations.

	**Time steps**	**Regularization parameter [*µ*]**	**Regularization parameter [*λ*]**
**Iteration 1**	1	4	1
**Iteration 2**	1	2	1
**Iteration 3**	2	1	.5
**Iteration 4**	4	.5	.25
**Iteration 5**	16	.25	.125
**Iteration 6**	64	.25	.125

**Fig. 2:**
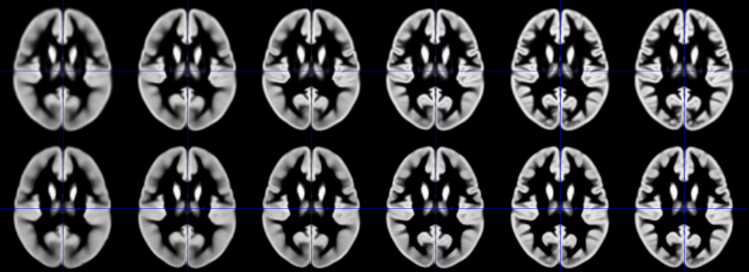
Top row: Illustration of the conventionally generated DARTEL GM templates, from the whole dataset (*n*=1919). Bottom row: Illustration of synthetically-generated DARTEL GM templates, generated by the CerebroMatic toolbox based on the here-presented regression parameter set (settings: age=330 months, field strength=3 T, gender=male, data quality=best).

**Fig. 3:**
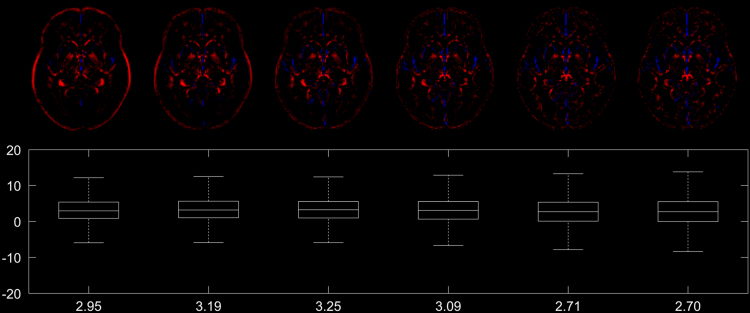
Top row: difference image of the conventionally generated and the synthetic DARTEL GM templates (cf. Fig. 2), showing voxels where the intensity difference exceeds 5% (in red) or −5% (in blue). Note overall only minor and decreasing differences. Bottom row: boxplot of all voxelwise differences, with the mean voxelwise intensity difference listed at the bottom (in %).

**Fig. 4:**
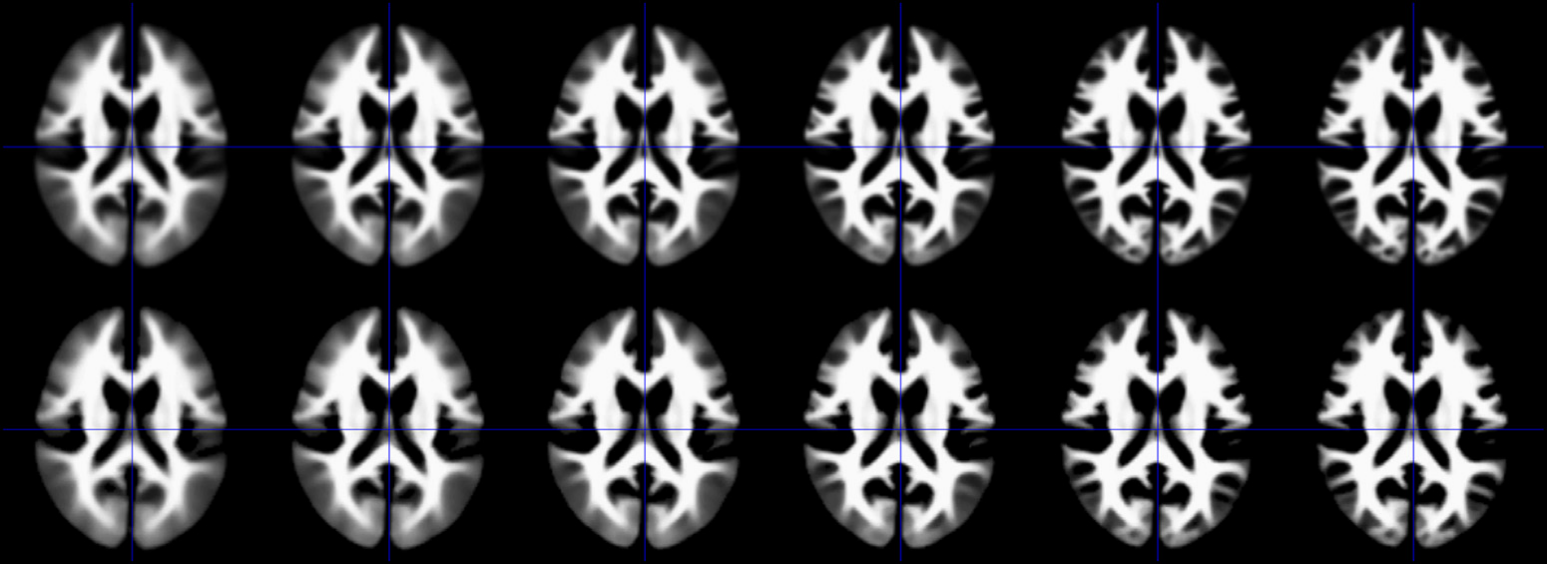
Top row: Illustration of the conventionally generated DARTEL WM templates, from the whole dataset (*n*=1919). Bottom row: Illustration of synthetically-generated DARTEL WM templates, generated by the CerebroMatic toolbox based on the here-presented regression parameter set (settings: age=330 months, field strength=3 T, gender=male, data quality=best).

**Fig. 5:**
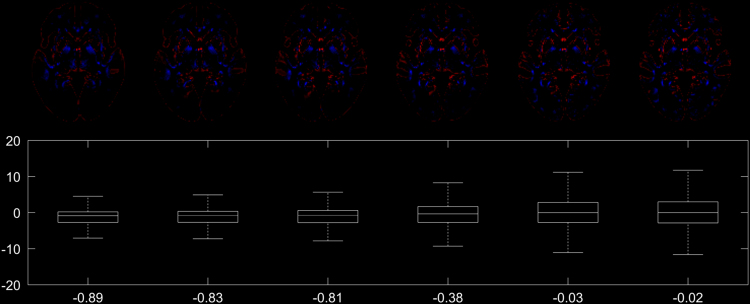
Top row: difference image of the conventionally generated and the synthetic DARTEL WM templates (cf. Fig. 4), showing voxels where the intensity difference exceeds 5% (in red) or −5% (in blue). Note overall only very minor and decreasing differences. Bottom row: boxplot of all voxelwise differences, with the mean voxelwise intensity difference listed at the bottom (in %).

Image data analysis was performed within the CerebroMatic toolbox which employs a multivariate adaptive regression spline approach as available within the ARESLab toolbox [20]. The data analysis settings were left at the defaults described in [1]. Due to their dominating influence, we used age and gender [2], [21], [22], as well as field strength [4] and data quality [3] as predictors. The latter was here described by the cat12 overall image quality measure, a combined parameter with contributions from spatial resolution, image noise, and image inhomogeneity [16]. Processing each iteration required about 12 hours per tissue class on a current PC workstation. The resulting regression parameters can now be used to generate a set of six increasingly crisp tissue maps (see Fig. 2, Fig. 4 for an illustration of the tissue maps, and Fig. 3, Fig. 5 for an illustration of their respective differences), matched to the demographic and technical details of a population under study, with regard to age (in the range of 13–900 months [1–75 years]), gender (male or female), and field strength (1.5 or 3 T). Tissue quality will automatically be set to “best”. These tissue maps can then serve as appropriately matched targets for spatial normalization within the DARTEL/SHOOT framework even for smaller studies, or studies of “unusual” populations.

## Footnotes

1 For this latter (SHOOT) approach, the resulting multi-volume tissue prior needs to be modified in that an additional volume is added that ensures that tissue probabilities sum to one [9], which can be done post-hoc. Hence, the same regression parameters for the two main tissue classes can be used for both approaches.
