# Supplementary material for: A spline-based regression parameter set for creating customized DARTEL MRI brain templates from infancy to old age
Source: Data Brief. 2017 Dec 12;16:959–66. doi: 10.1016/j.dib.2017.12.001 (PMC5752094; doi:10.1016/j.dib.2017.12.001)
Supplement: Supplementary file 1 — Supplementary material [file mmc1.pdf]

Data Article

A spline-based regression parameter set for creating customized DARTEL MRI brain templates from infancy to old age

Marko Wilke

Transparency statement:

This study was funded in part by a grant from the H.W. & J. Hector Foundation, Mannheim (M66). The sponsor had no role in study design, in the collection, analysis and interpretation of data, in the writing of the report, and in the decision to submit the article for publication. I also acknowledge support by Deutsche Forschungsgemeinschaft and Open Access Publishing Fund of University of Tübingen.
